# miR-125b and miR-100 Are Predictive Biomarkers of Response to Induction Chemotherapy in Osteosarcoma

**DOI:** 10.1155/2016/1390571

**Published:** 2016-11-21

**Authors:** Daisuke Kubota, Nobuyoshi Kosaka, Tomohiro Fujiwara, Akihiko Yoshida, Yasuhito Arai, Zhiwei Qiao, Fumitaka Takeshita, Takahiro Ochiya, Akira Kawai, Tadashi Kondo

**Affiliations:** ^1^Division of Rare Cancer Research, National Cancer Center Research Institute, 5-1-1 Tsukiji, Chuo-ku, Tokyo 104-0045, Japan; ^2^Division of Musculoskeletal Oncology, National Cancer Center Hospital, 5-1-1 Tsukiji, Chuo-ku, Tokyo 104-0045, Japan; ^3^Division of Molecular and Cellular Medicine, National Cancer Center Research Institute, 5-1-1 Tsukiji, Chuo-ku, Tokyo 104-0045, Japan; ^4^Pathology and Clinical Laboratory Division, National Cancer Center Hospital, 5-1-1 Tsukiji, Chuo-ku, Tokyo 104-0045, Japan; ^5^Division of Cancer Genomics, National Cancer Center Research Institute, 5-1-1 Tsukiji, Chuo-ku, Tokyo 104-0045, Japan; ^6^Department of Functional Analysis, National Cancer Center Research Institute, 5-1-1 Tsukiji, Chuo-ku, Tokyo 104-0045, Japan

## Abstract

Osteosarcoma is the most common primary malignancy in bone. Patients who respond poorly to induction chemotherapy are at higher risk of adverse prognosis. The molecular basis for such poor prognosis remains unclear. We investigated miRNA expression in eight open biopsy samples to identify miRNAs predictive of response to induction chemotherapy and thus maybe used for risk stratification therapy. The samples were obtained from four patients with inferior necrosis (Huvos I/II) and four patients with superior necrosis (Huvos III/IV) following induction chemotherapy. We found six miRNAs, including miR-125b and miR-100, that were differentially expressed > 2-fold (*p* < 0.05) in patients who respond poorly to treatment. The association between poor prognosis and the abundance of miR-125b and miR-100 was confirmed by quantitative reverse transcriptase-polymerase chain reaction in 20 additional osteosarcoma patients. Accordingly, overexpression of miR-125b and miR-100 in three osteosarcoma cell lines enhanced cell proliferation, invasiveness, and resistance to chemotherapeutic drugs such as methotrexate, doxorubicin, and cisplatin. In addition, overexpression of miR-125b blocked the ability of these chemotherapy agents to induce apoptosis. As open biopsy is routinely performed to diagnose osteosarcoma, levels of miR-125b and miR-100 in these samples may be used as basis for risk stratification therapy.

## 1. Introduction

Osteosarcoma is the most common primary malignancy in bone and a leading cause of cancer death among children and adolescents [1, 2]. Cure rates of 15–20% were achieved in the 1970s by surgery alone in patients with localized osteosarcoma. These rates dramatically improved to as high as 80% following the introduction of higher-dose and multiagent chemotherapy regimens and induction chemotherapy [3, 4]. Induction chemotherapy downstages tumors and facilitates complete resection by inhibiting micrometastatic tumors and decreasing tumor vascularity. Response to induction chemotherapy is histologically evaluated according to the Huvos grading system [5], which is based on the degree of tumor necrosis in surgically resected tissues. Patients with ≥90% tumor necrosis after induction chemotherapy are considered good responders, and all others are deemed to be poor responders [2]. It is noteworthy that histological response to induction chemotherapy is the most reliable prognostic factor, aside from metastasis at time of diagnosis [6–15]. Therefore, prediction of response to induction chemotherapy could potentially be used to determine the most appropriate treatment regimen [16].

Although Huvos grading is widely used, it is obtained after chemotherapy and is thus not predictive. On the other hand, clinically useful predictive biomarkers have not been identified, even though osteosarcoma has been extensively characterized. This has prevented effective stratification of patients according to risk of drug resistance and may prevent further innovations in treatment. Therefore, it is imperative to understand the molecular basis of chemoresistance to develop more effective therapies.

Osteosarcoma is genetically heterogeneous among patients, across tumors, and within tumors [17, 18]. Indeed, osteosarcoma karyotypes indicate numerous numerical and structural changes [19]. Therefore, a comprehensive omics approach to survey molecular events at multiple levels may identify novel molecular mechanisms underlying resistance to treatments. Given the complex mechanisms that can contribute to chemoresistance, significant biological insights may yet be uncovered.

Previously, we investigated the proteomic profiles of open biopsy samples obtained from osteosarcoma patients before chemotherapy and identified peroxiredoxin 2 (PRDX2) as a novel predictive biomarker with response to induction chemotherapy with ifosfamide, doxorubicin, and cisplatin [20]. Subsequently, we found PRDX2 to be also predictive of the response to induction chemotherapy with different combinations of drugs, and we characterized its functional significance [21]. As open biopsy is routinely performed to diagnose osteosarcoma, predictive biomarkers that can be measured in samples collected during this procedure may prove to be useful in clinical settings.

microRNAs (miRNAs) are small, noncoding RNA 21–25 nucleotides in length that control growth, development, and differentiation by regulating gene expression posttranscriptionally. The human genome encodes more than 1,000 miRNAs [22] that regulate thousands of human genes [23, 24]. In osteosarcoma, global expression of miRNAs has been examined in relation to onset [25, 26], progression [27, 28], response to treatments [29, 30], and prognosis [31]. However, the clinical significance of these miRNAs has not been definitively established.

In this study, we explored the possibility that expression of miRNAs may have a utility in predicting responsiveness to neoadjuvant chemotherapy in osteosarcoma patients. We analyzed miRNA expression in frozen tissue samples obtained before induction chemotherapy. We found that abundant expression of miR-125b and miR-100 was significantly associated with poor response to chemotherapy. We validated this result using qRT-PCR in an independent sample set and verified the functional significance of these miRNAs by* in vitro* assays.

## 2. Material and Methods

### 2.1. Patients and Clinical Information

Frozen clinical specimens, collected by open biopsy before chemotherapy, were retrieved from the National Cancer Center Hospital, Japan. The samples were obtained from eight patients (Table 1) who were diagnosed between 2009 and 2011 and treated according to the standard treatment protocol with methotrexate, doxorubicin, and cisplatin, which are considered key drugs [32]. Response to preoperative chemotherapy was histologically assessed by a pathologist according to the Huvos grading system [2]; when less than 10% tumor cells were found to be viable, the patients were defined as responders, and if not, they were considered as nonresponders (chemoresistant). Samples were snap-frozen in liquid nitrogen at the time of collection and stored at −80°C.

An independent cohort of 20 patients (Table 2) was used to validate results by qRT-PCR, using formalin-fixed paraffin-embedded specimens also collected by open biopsy before chemotherapy. These patients were diagnosed between 1990 and 2008. Nine patients were chemoresistant, and 11 were chemosensitive. All patients were monitored from 0.5 to 11.1 years, with mean 7.7 years. Seven patients have been continuously disease-free, seven died of the disease, and six were living with osteosarcoma.

Written informed consent was obtained from all patients, and the study was approved by the ethics committee of the National Cancer Center.

### 2.2. RNA Extraction and miRNA and mRNA Array

Frozen tissues were powdered under liquid nitrogen using Multi-beads shocker (Yasui Kikai, Osaka, Japan). Total RNA was then extracted using miRNeasy Mini Kit (Qiagen, Venlo, Netherlands). Formalin-fixed paraffin-embedded samples were sectioned at 10 *μ*m, and total RNA was extracted from several of such slices using the miRNeasy FFPE Kit (Qiagen).

miRNA expression profiles of frozen samples were obtained by hybridizing total RNA to the Agilent human miRNA Microarray V3 (021827, 8 × 15 K, v12.0, Agilent Technologies, Santa Clara, CA), following the manufacturer's instructions. Hybridized microarrays were scanned with a DNA microarray scanner (Agilent G2565BA) with default protocols and settings. miRNA expression was analyzed in GeneSpring GX software (Agilent Technologies). miRNAs were considered differentially expressed when expression increased or decreased > 1.5-fold, with *p* < 0.05 in an unpaired *t*-test.

mRNA expression profiles of osteosarcoma cells were obtained by hybridizing total RNA to SurePrint G3 Human GE microarray (8 × 60 K, Ver3.0, Agilent Technologies, Santa Clara, CA), following the manufacturer's instructions. The DNA microarray data were analyzed by Bioconductor agilp package (http://bioconductor.org/packages/agilp/). The probes were selected, when their intensity was considerably (more than 1.5-fold change) different between two samples.

### 2.3. Validation of miR-100 and miR-125b in Independent Samples

miR-100 and miR-125b were amplified by quantitative real-time reverse transcriptase-polymerase chain reaction (qRT-PCR) from formalin-fixed paraffin-embedded samples. First, cDNA was synthesized from 10 ng total RNA using TaqMan MicroRNA hsa-miR-100 and hsa-miR-125b (Applied Biosystems) and TaqMan MicroRNA Reverse Transcription Kit (Applied Biosystems). Target miRNAs were amplified over 45 cycles of denaturation at 95°C for 10 sec, annealing at 60°C for 10 sec, and extension at 65°C for 10 sec, following initial denaturation at 95°C for 10 min. PCR was performed in triplicate in 96-well plates using the 7300 Real-Time PCR System (Applied Biosystems), and miRNA expression was normalized to small nuclear RNA RNU6B (Applied Biosystems).

### 2.4. Cell Culture and Transfection

Differentially expressed miRNAs were functionally characterized in osteosarcoma cell lines MNNG-HOS, 143B, and MG63, American Type Culture Collection. Briefly, cells were cultured at 37°C in a humidified 5% CO_2_ chamber using DMEM supplemented with 10% fetal bovine serum, 1 mmol/L sodium pyruvate, nonessential amino acids, and 2 mmol/L glutamine. Cells were then transfected with 25 nM negative control miRNA (Cosmo Bio, Tokyo, Japan), miR-100 (Bonac Corporation, Fukuoka, Japan), and miR-125 (Bonac Corporation, Fukuoka, Japan) using DharmaFECT transfection reagents (Thermo Scientific, Tokyo, Japan), following the manufacturer's protocol.

### 2.5. Cell Proliferation Assay

Cell proliferation was measured using a standard MTS assay. In brief, cells were seeded at 3 × 10^3^ cells/well in a 96-well plate containing 100 *μ*L culture medium, grown overnight, and transfected with miRNAs as described or incubated in conditioned medium. The media in transfected cells were replaced with fresh medium A 24 h after transfection. Cell viability was determined 3 d thereafter by Cell Counting Kit-8 (Dojindo, Kumamoto, Japan) according to the manufacturer's protocol. In this assay, WST-8 is reduced by dehydrogenase activity in viable cells to yield a formazan dye, which is soluble in tissue culture media, and assayed by absorbance at 450 nm [33]. The amount of formazan dye was produced in directly proportional to the number of viable cells. Three wells were examined for each treatment, and experiments were independently repeated three times. Results are reported as mean ± SD. Statistical significance was tested by *t*-test.

### 2.6. Cell Invasion Assay

Cell invasion was evaluated using the BD BioCoat™ Invasion Chamber (BD Bioscience), following the manufacturer's protocol. In brief, cells transfected with miR-125b or miR-100 for 24 h were seeded onto the membrane in the upper chamber of the transwell at 5 × 10^5^ cells in 500 *μ*L serum-free medium. The medium in the lower chamber contained 10% fetal calf serum as source of chemoattractants. Cells that passed through the Matrigel-coated membrane were stained with Diff-Quick (Sysmex, Kobe, Japan) and counted. Experiments were performed three times independently, and statistical significance was determined by *t*-test.

### 2.7. Apoptosis Assay

Cells were transfected as described with 25 nM negative control miRNA, miR-100, and miR-125b and treated 24 h thereafter with methotrexate for 72 h, doxorubicin for 48 h, and cisplatin for 48 h. Proteins were extracted, and expression of apoptosis-associated proteins was measured by western blotting.

### 2.8. Protein Extraction and Western Blotting

Frozen tissues were powdered under liquid nitrogen with the Multi-beads shocker and resuspended in 6 mol/L urea, 2 mol/L thiourea, 3% CHAPS, and 1% Triton X-100. The supernatant was then cleared by centrifugation at 15,000 rpm for 30 min. Cultured cells were washed with PBS, fixed with 10% TCA for 30 min, and lysed in the same buffer for 30 min. The supernatant was collected after centrifugation for 30 min.

Proteins were separated on SDS-polyacrylamide gels and transferred to a nitrocellulose membrane (Bio-Rad). After blocking for 1 h in Tris-buffered saline and Tween 20 (TBS-T) supplemented with 5% nonfat milk, membranes were incubated overnight at 4°C with primary antibodies against Bak1 (monoclonal, 1 : 250, MBL), PARP (1 : 1000, BD), Caspase-3 (1 : 1000, BD), Caspase-9 (1 : 1000, MBL), Caspase-2 (1 : 1000, BD), BMF (polyclonal, 1 : 1000, MBL), PUMA (monoclonal, 1 : 1000, BD), Bcl-2 (monoclonal, 1 : 500, BD), PP2A-catalytic *α* (1 : 1000, BD), MCL-1 (1 : 1000, BD), and *β*-actin (1 : 1000, BD). Membranes were then extensively washed with TBS-T and labeled with horseradish peroxidase-conjugated secondary anti-mouse (1 : 1000, GE) or anti-rabbit IgG (1 : 2000, GE). After additional washes with TBS-T, antigen-antibody complexes were visualized with ECL-Prime Kit (GE).

### 2.9. Transfection of miRNA to Osteosarcoma Cells Followed by Global Gene Expression Analysis

For preparation of RNA samples, MG63 cells were transfected with 25 nM negative control miRNA, miR-100, and miR-125 using DharmaFECT transfection reagents. Cells were harvested at 72 hours after transfection. mRNA expression profiles of miRNA transfected cells were obtained for the global mRNA expression study using DNA microarray as mentioned above.

## 3. Results

**Figure 1:**
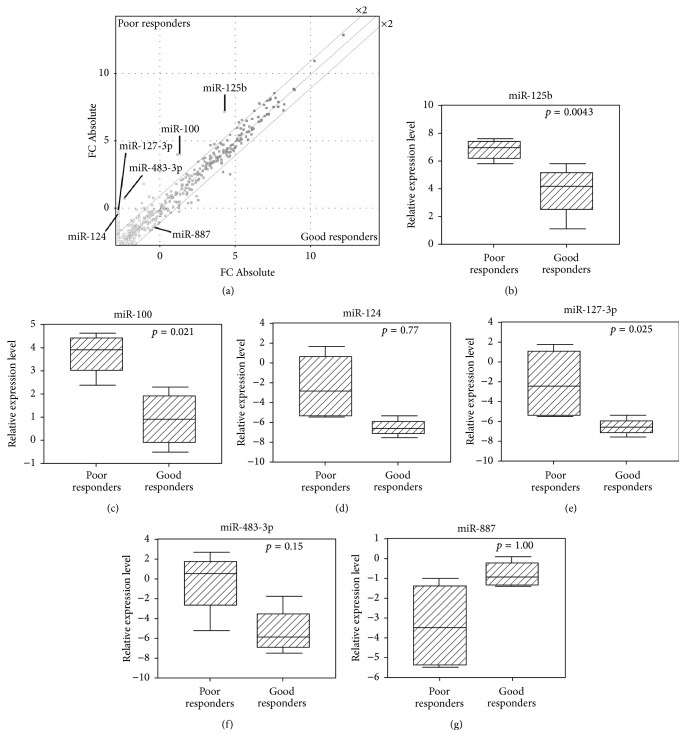
Expression of six miRNAs was significantly different between poor and good responders, as measured by microarray analysis of open biopsy samples (a). qRT-PCR (b–g) of these six miRNAs confirmed that miRNA-125b (b) and miR-100 (c) were expressed at significantly higher levels in chemoresistant patients.

We analyzed global miRNA expression in eight frozen diagnostic open biopsy specimens. Clinical and pathological data are summarized in Table 1. The tumors included in the miRNA expression study were histologically osteoblastic and originated from either the femur or the tibia. Since a previous study has suggested there was a significant correlation between response to chemotherapy and histology [34], we aimed to omit the histological bias in our study by using samples with similar histological and anatomical backgrounds. Patients who had less than 10% viable tumor cells after chemotherapy were considered good responders, and all others were deemed to be poor responders according to the Huvos grading system. Expression profiling identified six miRNAs expressed at significantly different levels (*p* < 0.05) between good responders and poor responders (Figure 1(a), Supplementary Table 1 in Supplementary Material available online at http://dx.doi.org/10.1155/2016/1390571). Of these, the miRNAs 483-3p, 100, 124, 125b, and 127-3p were expressed more abundantly in poor responders, while miR-887 expression was suppressed. Clinical or pathological factors were not obviously correlated with differential expression. To confirm results, these miRNAs were amplified by qRT-PCR from the same eight samples. This assay confirmed significantly different (*p* < 0.05) expression of miR-125b and miR-100 in poor responders (Figures 1(b) and 1(c)) but not of miRNAs 124, 127-3p, 483-3p, and 887 (Figures 1(d)–1(g)). Nevertheless, expression levels measured by qRT-PCR show the same general trends as microarray data.

**Figure 2:**
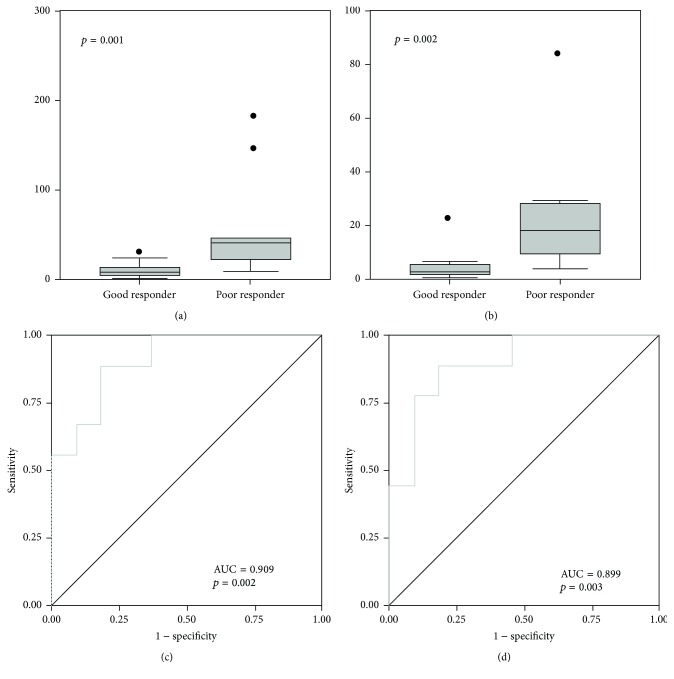
In an independent cohort of patients, expression of miR-125b (a) and miR-100 (b) was higher in patients resistant induction chemotherapy. miR-125b (c) and miR-100 (d) showed significant sensitivity and specificity in receiver operating curves, with area under the curve 0.909 and 0.899, respectively. The black circles represent the patients' data.

To investigate these observations further, we measured miRNA expression by qRT-PCR in 20 formalin-fixed paraffin-embedded open biopsy samples of primary osteosarcoma (Supplementary Table 2). The tumor tissues used for validation purposes were heterogeneous; they included osteoblastic, chondroblastic, or fibroblastic tumors, and their site of origin included the tibia, the femur, or the ilium. By exploring such heterogeneous tumor samples, we aimed to demonstrate the versatility of these miRs in predicting responsiveness to neoadjuvant chemotherapy. We also found miR-125b and miR-100 to be expressed at significantly higher levels (*p* < 0.05) in poor responders (Figures 2(a) and 2(b)). The receiver operating characteristic curve had areas under the curves 0.909 and 0.899 for miR-125b and miR-100, respectively (*p* < 0.05, Figures 2(c) and 2(d)).

**Figure 3:**
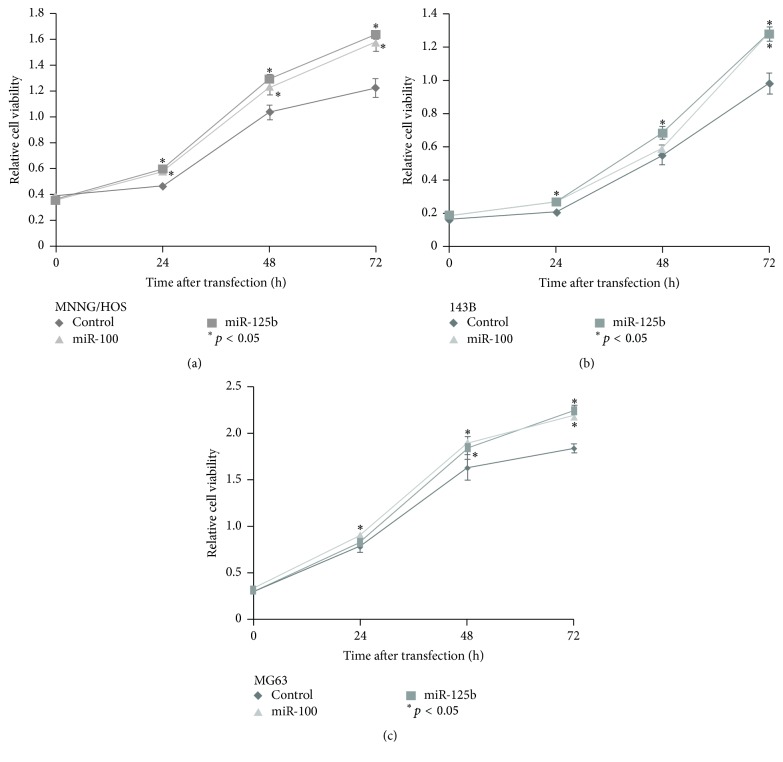
Transfection of miRNA-100 and miR-125b significantly enhanced (*p* < 0.05) proliferation in osteosarcoma cell lines MNNG/HOS (a), 143B (b), and MG63 (c).

**Figure 4:**
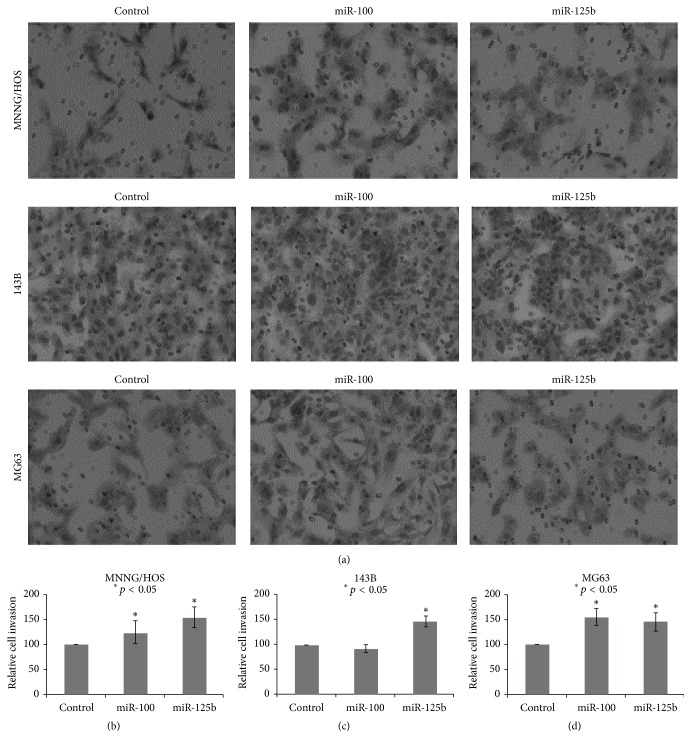
Transfection of miR-100 and miR-125b enhanced cell invasiveness in MNNG/HOS (a), 143B (b), and MG63 (c) osteosarcoma cell lines.

In light of these results, we characterized the functional significance of miR-125b and miR-100 overexpression in osteosarcoma cells. In cell proliferation assays, we observed miR-125b and miR-100 to significantly enhance growth in MNNG/HOS, 143B, and MG63 (Figures 3(a)–3(c)). Moreover, we found that miR-125b promoted cell invasiveness in MNNG/HOS and 143B, while miR-100 enhanced cell invasiveness in all three cell lines (Figures 4(a) and 4(b)). These observations suggested that overexpression of miR-125b and miR-100 had significant effects on tumorigenicity.

We investigated the impact of miR-125b and miR-100 on the effectiveness of chemotherapy drugs. Overexpression of miR-125b significantly blocked the cytotoxic effects of methotrexate, doxorubicin, and cisplatin in all cells (Supplementary Figure 1, Supplementary Table 3). miR-100 also blocked cytotoxicity, except that of doxorubicin in MNNG/HOS cells (Supplementary Figure 1, Supplementary Table 3). Subsequently, we focused on miR-125b because of its ability to block all chemotherapy drugs in all cells examined.

We hypothesized that the known ability of miR-125b to inhibit apoptosis drives tumor progression and resistance to chemotherapy. Indeed, we found that overexpression of miR-125b markedly reduced the expression of apoptosis proteins, including p53, Caspase-2, MCL-1, PUMA, and PP2A catalytic *α* (Supplementary Figure 2(A)). We also found that miR-125b blocked the ability of methotrexate, doxorubicin, and cisplatin to induce expression of Bak1 and cleaved PARP (Supplementary Figure 2(B)). On the other hand, overexpression of miR-100 resulted in reduced expression of CTDSPL and Rb (Supplementary Figure 2(C)). These results are consistent with our hypothesis that miR-125b may contribute to drug resistance by inhibiting apoptosis.

To explore the effects of miR-100 and miR-125b, we examined the global expression of mRNA after transfecting miR-100 or miR-125b. The transfection of miR-100 and miR-125b induced the differential expression of 35 and 88 genes, respectively (Supplementary Tables 4 and 5). The 16 mRNAs were commonly up- or downregulated by transfection of miR-100 and miR-125b (Table 3).

## 4. Discussion

As osteosarcoma patients have significantly favorable prognosis when they respond well to neoadjuvant treatments, molecular biomarkers predictive of this response may be useful to determine the appropriate course of treatment. Several of such biomarkers have been reported, including P-glycoprotein [15] and PRDX2, which was associated with resistance to neoadjuvant treatments in two independent studies [20, 21]. In contrast, Borys et al. [35] reported that p16 was correlated with a therapeutic response. Although these studies concerning miR-100 and miR-125b appear promising, there is insufficient evidence to support their practical use. Extensive validation studies will be required to establish the predictive utility of these biomarkers prior to application in the clinic.

We now report that abundant expression of miR-100 and miR-125b in osteosarcoma tissues prior to treatment is predictive of a poor response to neoadjuvant chemotherapy. Notably, expression of miR-100 and miR-125b was found to be lower in osteosarcoma tissues than in adjacent healthy tissues [36, 37]. As we examined expression of these miRNAs only in tumor tissues, it might be informative to investigate whether expression in adjacent tissues is correlated in some way with clinical and pathological parameters. The superiority of miR-100 and miR-125b over other previously reported predictive biomarkers should be noted. The area under the ROC curve for PRDX2 has been reported to be 0.90 (*p* = 0.015) [20, 21]. The predictive performances of miR-100 and miR-125b noted here (areas under the ROC curve of approximately 0.9 in each case) are equivalent to that of PRDX2. However, the expression of PRDX2 was measured by western blotting, a methodology that cannot be easily automated. In contrast, the expression levels of miR-100 and miR-125b were measured by qRT-PCR, which can be easily automated. The practical utility of miR-100 and miR-125b should therefore be further explored.

The reported effects of miR-100 and miR-125b on proliferation of osteosarcoma cells are controversial. Huang et al. [36] demonstrated that miR-100 suppresses proliferation of Saos-2 and MG63 cells, an effect reversed by antisense RNA against miR-100. In contrast, we found miR-100 as well as miR-125b to stimulate proliferation in MNNB/HOS, 143B, and MG63 cells (Figure 3). A possible explanation for this discrepancy is the use of different assays to measure cell proliferation [33]. As osteosarcoma is a heterogeneous malignancy, in order to establish the molecular mechanisms of chemoresistance we may need to explore a range of osteosarcoma cell lines.

We found the mRNAs expression which was commonly upregulated by the transfection of miR-100 and miR-125b. Among them, the expression of sirtuin (silent mating type information regulation 2 homolog 5, SIRT5) was previously associated with the resistance against the* in vitro* and* in vivo* treatments with cis-diamminedichloroplatinum, 5-fluorouracil, or bleomycin in non-small cell lung cancer [38]. The aberrant regulation of SIRT5 was reported in various types of malignancies. For example, in breast cancer, the expression of SIRT5 had significant relation with tumor location, grade, and expression of estrogen receptor or progesterone receptor [39]. However, the aberrant expression of SIRT5 regulated by miRNAs was not implicated with osteosarcoma until our study. The sirtuin family genes play an important role in the carcinogenesis and cancer progression [40], and it is worth challenging to investigate the regulation and functions of sirtuins in osteosarcomas.

Our next challenge is to establish the clinical utility of miR-100 and miR-125b. As the number of cases in this study was limited, more validation studies may be required before clinical application. Prediction of a poor response to neoadjuvant chemotherapy may prevent the use of time-consuming but ultimately ineffective treatments and thereby prevent patients from experiencing unnecessary side effects. Unfortunately, there are no alternative therapeutic strategies. Nevertheless, immediate surgical resection due to a predicted risk of chemoresistance may improve clinical outcomes for some patients. Identification of chemoresistant patients may also justify their inclusion in clinical trials of novel anticancer agents. Finally, we may be able to identify new therapeutic targets by characterizing the molecular basis of the correlation between miR-100 and miR-125b expression and response to neoadjuvant chemotherapy.

The global expression of miRNAs and especially the expression level of miR-100 and miR-125b in metastatic tumors is intriguing. Recently, Berlanga et al. compared primary osteosarcomas with lung metastatic osteosarcomas and identified twenty-six miRNAs with significantly different expression between the two [41]. Although miR-100 and miR-125b were not identified in their study, it is worth examining their expression in paired primary and metastatic tumor tissues in patients having different responses to chemotherapeutic treatment. The correlation between the miR-100 and miR-125b expression levels and tumor recurrence is also worth exploring. Sanchez-Diaz et al. have previously reported the identity of miRNAs associated with tumor recurrence in pediatric osteosarcoma [42]. Probably because they focused on pediatric osteosarcoma, miR-100 and miR-125b were not identified as being associated with tumor recurrence. As the response to neoadjuvant chemotherapy is associated with a favorable prognosis, the expression of miR-100 and miR-125b may be associated with poor prognosis in osteosarcoma. This hypothesis should be addressed in a future study.

In summary, expression of miR-100 and miR-125b in pretreatment of osteosarcoma is significantly correlated with poor chemotherapy response and is a promising biomarker to guide treatment decisions.

## Supplementary Material

List of supplementary materials
Supplementary List
Supplementary Figure 1
Effects of expression of miR-100 and miR-125b on drug sensitivity
Supplementary Figure 2
Effects of miR-100 and miR-125b on apoptosis
Supplementary Table 1
Expression data of six miRNAs by microarray
Supplementary Table 2
Expression data of six miRNAs by qRT-PCR
Supplementary Table 3
miRNAs changed sensitivity to chemotherapy drugs
Supplementary Table 4
Genes the expression of which was affected by miRNA-100 transfection
Supplementary Table 5
Genes the expression of which was affected by miRNA-125b transfection
Legends of supplementary figures
Supplementary Figure 1
Effects of expression of miR-100 and miR-125b on drug sensitivity. Expression of miR-100 and miR-125b was silenced in MNNG/HOS (A, B, C), 123B (D, E, F), and MG63 (G, H, I) cells, which were then exposed to methotrexate (A, D, G), doxorubicin (B, E, H), and cisplatin (C, F, I). IC_50_ values are summarized in Supplementary Table 3.
Supplementary Figure 2
Effects of miR-100 and miR-125b on apoptosis. Expression of apoptosis proteins was measured before and after transfection with miRNA-125b (A). Over-expression of miRNA-125b decreased expression of Bak1, p53, Caspase-2, MCL-1, PUMA, and PP2A catalytic α (A). miRNA-125b partially blocked the ability of methotrexate (MTX), doxorubicin (DOX), and cisplatin (CDDP) to suppress expression of Bak1 (B, upper panel), and induce cleaved PARP (B, middle panel). On the other hand, miR-100 inhibited the Rb pathway (C).

## Figures and Tables

**Table 1:** Clinical and pathological data of osteosarcoma patients for global miRNA expression study.

Case number	Gender	Age	Location	Histologic type	Neoadjuvant chemotherapy	Viable cells (%)^A^
Case 1	Female	11	Tibia	Osteoblastic	MTX + DOX + CDDP	0%
Case 2	Male	18	Tibia	Osteoblastic	MTX + DOX + CDDP	9%
Case 3	Male	15	Tibia	Osteoblastic	MTX + DOX + CDDP	0%
Case 4	Female	13	Femur	Osteoblastic	MTX + DOX + CDDP	0%
Case 5	Female	10	Femur	Osteoblastic	MTX + DOX + CDDP	70%
Case 6	Male	25	Tibia	Osteoblastic	MTX + DOX + CDDP	40%
Case 7	Male	12	Femur	Osteoblastic	MTX + DOX + CDDP	20%
Case 8	Female	16	Femur	Osteoblastic	MTX + DOX + CDDP	22%

A: probability of viable cells was pathologically evaluated by surgical specimen.

**Table 2:** Clinical and pathological data of osteosarcoma patients for validation study.

Case number	Gender	Age	Location	Histologic type	Neoadjuvant chemotherapy	Viable cells (%)^A^	Prognosis^C^
Number 1	Male	19	Tibia	Osteoblastic	MTX + DOX + CDDP	0–10%	NED
Number 2	Female	9	Femur	Osteoblastic	MTX + DOX + CDDP	0%	CDF
Number 3	Female	9	Tibia	Chondroblastic	MTX + DOX + CDDP	0–10%	DOD
Number 4	Female	14	Femur	Osteoblastic	MTX + DOX + CDDP	0	CDF
Number 5	Female	15	Femur	Osteoblastic	MTX + DOX + CDDP	0–10%	DOD
Number 6	Female	9	Tibia	Osteoblastic	MTX + DOX + CDDP	0%	NED
Number 7	Male	17	Femur	Osteoblastic	MTX + DOX + CDDP	0–10%	CDF
Number 8	Female	17	Tibia	Osteoblastic	MTX + DOX + CDDP	1%	CDF
Number 9	Female	11	Femur	Chondroblastic	MTX + DOX + CDDP	7%	CDF
Number 10	Male	13	Femur	Osteoblastic	MTX + DOX + CDDP	0–10%	DOD
Number 11	Male	16	Femur	Chondroblastic	MTX + DOX + CDDP	0–10%	DOD
Number 12	Female	13	Tibia	Osteoblastic	MTX + DOX + CDDP	60–70%	DOD
Number 13	Male	22	Femur	Osteoblastic	MTX + DOX + CDDP	30%	DOD
Number 14	Male	18	Femur	Osteoblastic	MTX + DOX + CDDP	60–70%	NED
Number 15	Male	14	Tibia	Osteoblastic	MTX + DOX + CDDP	50–60%	CDF
Number 16	Male	13	Femur	Osteoblastic	MTX + DOX + CDDP	30–40%	AWD
Number 17	Male	9	Femur	Chondroblastic	MTX + DOX + CDDP	30–40%	NED
Number 18	Female	10	Femur	Chondroblastic	MTX + DOX + CDDP	70%	CDF
Number 19	Male	17	Ilium	Fibroblastic	MTX + DOX + CDDP	Clinically poor^B^	DOD
Number 20	Male	15	Tibia	Osteoblastic	MTX + DOX + CDDP	50–60%	NED

A: Probability of viable cells was pathologically evaluated by surgical specimen. B: patients with disease progression in diagnostic imaging by computed tomography and magnetic resource imaging. C: CDF: continuously disease-free, DOD: died of disease, NED: no evidence of disease, and AWD: alive with disease.

**Table 3:** Genes commonly regulated by miRNA-100 and miRNA-125b.

Accession number	Symbol	Gene title	Biological function
*Upregulated genes*			
NM_006868	RAB31	RAB31, member RAS oncogene family	Nucleotide binding
NM_012241	SIRT5	Sirtuin (silent mating type information regulation 2 homolog) 5	NAD+ ADP-ribosyltransferase activity
NM_001142864	PIEZO1	Piezo-type mechanosensitive ion channel component 1	Cation channel activity
NM_002383	MAZ	MYC-associated zinc finger protein	Metal ion binding
NM_004997	MYBPH	Myosin binding protein H	Structural constituent of muscle
NM_000261	MYOC	Myocilin, trabecular meshwork inducible glucocorticoid response	Structural molecule activity
NM_014292	CBX6	Chromobox homolog 6	Chromatin binding
NM_145267	C6orf57	Chromosome 6 open reading frame 57	Unknown

*Downregulated genes*			
NM_000576	IL1B	Interleukin 1, beta	Cytokine activity
NM_014510	PCLO	piccolo (presynaptic cytomatrix protein)	Calcium ion binding
NM_001017402	LAMB3	Laminin, beta 3	Structural molecule activity
NM_004864	GDF15	Growth differentiation factor 15	Cytokine activity
NM_000379	XDH	Xanthine dehydrogenase	Nucleotide binding
NM_182507	KRT80	Keratin 80	Structural molecule activity
NM_213602	SIGLEC15	Sialic acid binding Ig-like lectin 15	Unknown
NM_005565	LCP2	Lymphocyte cytosolic protein 2	Unknown
